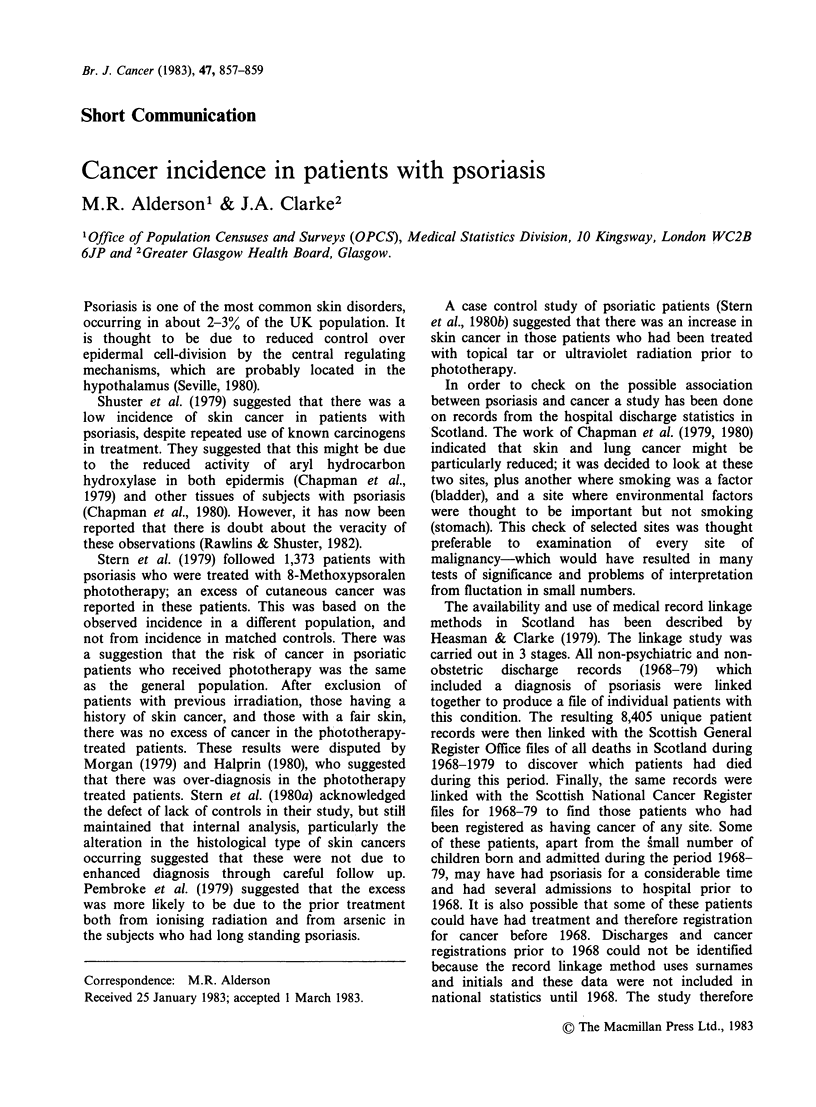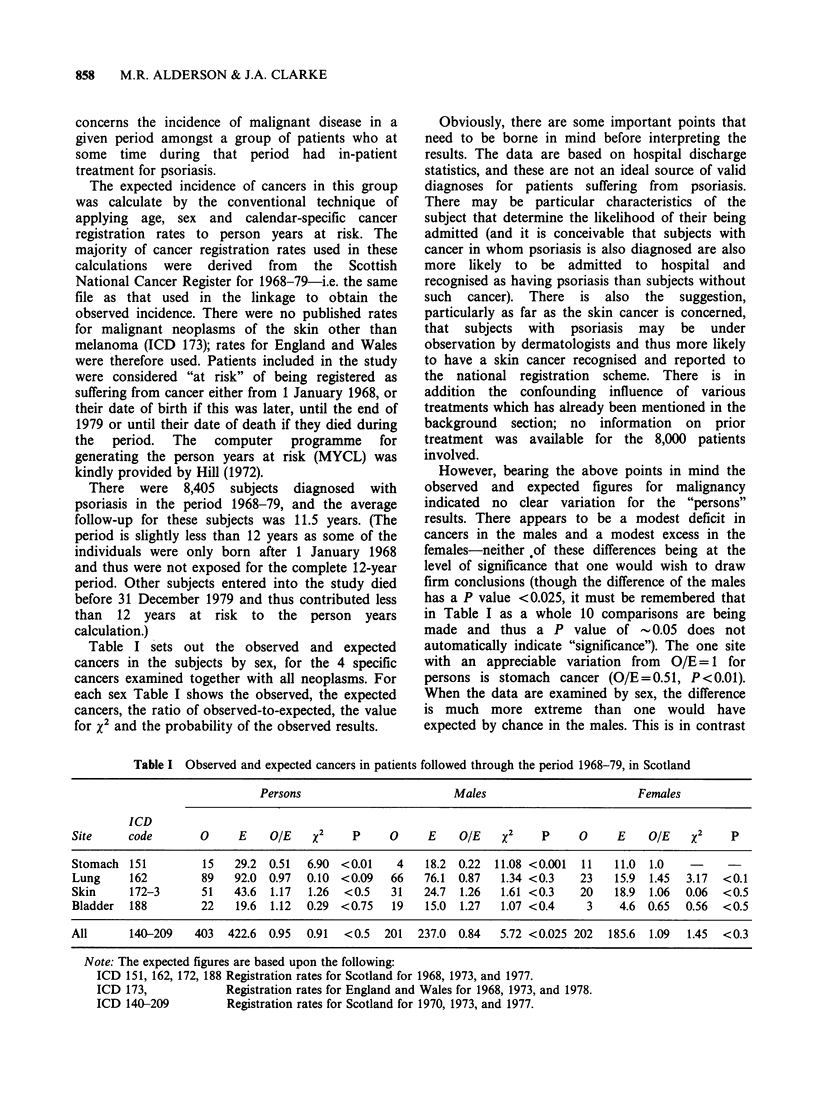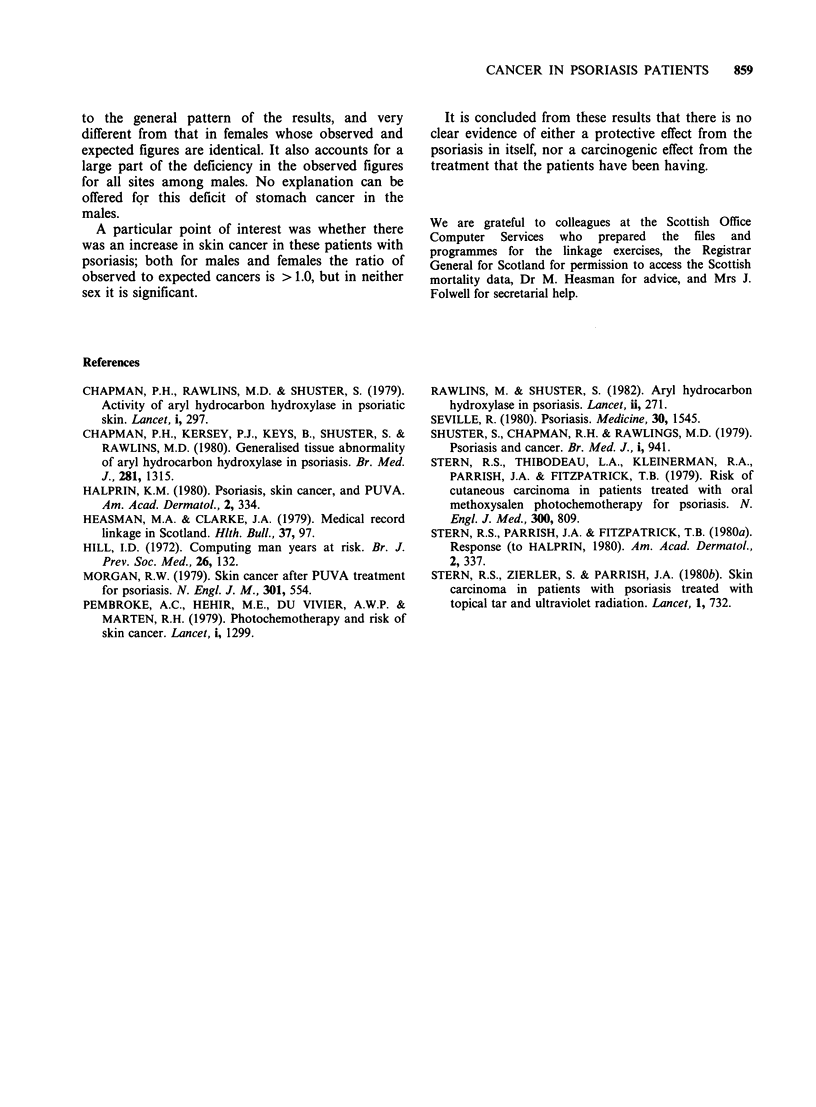# Cancer incidence in patients with psoriasis.

**DOI:** 10.1038/bjc.1983.142

**Published:** 1983-06

**Authors:** M. R. Alderson, J. A. Clarke


*Br. J. Cancer* (1983), **47**, 857–859

### Short Communication

### Cancer incidence in patients with psoriasis

M.R. Alderson[1] & J.A. Clarke[2]

[1]*Office of Population Censuses and Surveys (OPCS), Medical Statistics Division, 10 Kingsway, London WC2B 6JP and* [2]*Greater Glasgow Health Board, Glasgow.*

Psoriasis is one of the most common skin disorders, occurring in about 2–3% of the UK population. It is thought to be due to reduced control over epidermal cell-division by the central regulating mechanisms, which are probably located in the hypothalamus (Seville, 1980).

Shuster *et al.* (1979) suggested that there was a low incidence of skin cancer in patients with psoriasis, despite repeated use of known carcinogens in treatment. They suggested that this might be due to the reduced activity of aryl hydrocarbon hydroxylase in both epidermis (Chapman *et al.*, 1979) and other tissues of subjects with psoriasis (Chapman *et al.*, 1980). However, it has now been reported that there is doubt about the veracity of these observations (Rawlins & Shuster, 1982).

Stern *et al.* (1979) followed 1,373 patients with psoriasis who were treated with 8-Methoxypsoralen phototherapy; an excess of cutaneous cancer was reported in these patients. This was based on the observed incidence in a different population, and not from incidence in matched controls. There was a suggestion that the risk of cancer in psoriatic patients who received phototherapy was the same as the general population. After exclusion of patients with previous irradiation, those having a history of skin cancer, and those with a fair skin, there was no excess of cancer in the phototherapy-treated patients. These results were disputed by Morgan (1979) and Halprin (1980), who suggested that there was over-diagnosis in the phototherapy treated patients. Stern *et al.* (1980*a*) acknowledged the defect of lack of controls in their study, but still maintained that internal analysis, particularly the alteration in the histological type of skin cancers occurring suggested that these were not due to enhanced diagnosis through careful follow up. Pembroke *et al.* (1979) suggested that the excess was more likely to be due to the prior treatment both from ionising radiation and from arsenic in the subjects who had long standing psoriasis.

A case control study of psoriatic patients (Stern *et al.*, 1980*b*) suggested that there was an increase in skin cancer in those patients who had been treated with topical tar or ultraviolet radiation prior to phototherapy.

In order to check on the possible association between psoriasis and cancer a study has been done on records from the hospital discharge statistics in Scotland. The work of Chapman *et al.* (1979, 1980) indicated that skin and lung cancer might be particularly reduced; it was decided to look at these two sites, plus another where smoking was a factor (bladder), and a site where environmental factors were thought to be important but not smoking (stomach). This check of selected sites was thought preferable to examination of every site of malignancy—which would have resulted in many tests of significance and problems of interpretation from fluctation in small numbers.

The availability and use of medical record linkage methods in Scotland has been described by Heasman & Clarke (1979). The linkage study was carried out in 3 stages. All non-psychiatric and non-obstetric discharge records (1968–79) which included a diagnosis of psoriasis were linked together to produce a file of individual patients with this condition. The resulting 8,405 unique patient records were then linked with the Scottish General Register Office files of all deaths in Scotland during 1968–1979 to discover which patients had died during this period. Finally, the same records were linked with the Scottish National Cancer Register files for 1968–79 to find those patients who had been registered as having cancer of any site. Some of these patients, apart from the small number of children born and admitted during the period 1968–79, may have had psoriasis for a considerable time and had several admissions to hospital prior to 1968. It is also possible that some of these patients could have had treatment and therefore registration for cancer before 1968. Discharges and cancer registrations prior to 1968 could not be identified because the record linkage method uses surnames and initials and these data were not included in national statistics until 1968. The study therefore

Correspondence: M.R. Alderson

Received 25 January 1983; accepted 1 March 1983.

© The Macmillan Press Ltd., 1983

concerns the incidence of malignant disease in a given period amongst a group of patients who at some time during that period had in-patient treatment for psoriasis.

The expected incidence of cancers in this group was calculate by the conventional technique of applying age, sex and calendar-specific cancer registration rates to person years at risk. The majority of cancer registration rates used in these calculations were derived from the Scottish National Cancer Register for 1968–79—i.e. the same file as that used in the linkage to obtain the observed incidence. There were no published rates for malignant neoplasms of the skin other than melanoma (ICD 173); rates for England and Wales were therefore used. Patients included in the study were considered "at risk" of being registered as suffering from cancer either from 1 January 1968, or their date of birth if this was later, until the end of 1979 or until their date of death if they died during the period. The computer programme for generating the person years at risk (MYCL) was kindly provided by Hill (1972).

There were 8,405 subjects diagnosed with psoriasis in the period 1968–79, and the average follow-up for these subjects was 11.5 years. (The period is slightly less than 12 years as some of the individuals were only born after 1 January 1968 and thus were not exposed for the complete 12-year period. Other subjects entered into the study died before 31 December 1979 and thus contributed less than 12 years at risk to the person years calculation.)

Table I sets out the observed and expected cancers in the subjects by sex, for the 4 specific cancers examined together with all neoplasms. For each sex Table I shows the observed, the expected cancers, the ratio of observed-to-expected, the value for $\chi^2$ and the probability of the observed results.

Obviously, there are some important points that need to be borne in mind before interpreting the results. The data are based on hospital discharge statistics, and these are not an ideal source of valid diagnoses for patients suffering from psoriasis. There may be particular characteristics of the subject that determine the likelihood of their being admitted (and it is conceivable that subjects with cancer in whom psoriasis is also diagnosed are also more likely to be admitted to hospital and recognised as having psoriasis than subjects without such cancer). There is also the suggestion, particularly as far as the skin cancer is concerned, that subjects with psoriasis may be under observation by dermatologists and thus more likely to have a skin cancer recognised and reported to the national registration scheme. There is in addition the confounding influence of various treatments which has already been mentioned in the background section; no information on prior treatment was available for the 8,000 patients involved.

However, bearing the above points in mind the observed and expected figures for malignancy indicated no clear variation for the "persons" results. There appears to be a modest deficit in cancers in the males and a modest excess in the females—neither of these differences being at the level of significance that one would wish to draw firm conclusions (though the difference of the males has a $P$ value $<0.025$, it must be remembered that in Table I as a whole 10 comparisons are being made and thus a $P$ value of $\sim 0.05$ does not automatically indicate "significance"). The one site with an appreciable variation from O/E = 1 for persons is stomach cancer (O/E = 0.51, $P<0.01$). When the data are examined by sex, the difference is much more extreme than one would have expected by chance in the males. This is in contrast

**Table I** Observed and expected cancers in patients followed through the period 1968–79, in Scotland

| | | *Persons* | | | | | *Males* | | | | | *Females* | | | | |
|---|---|---|---|---|---|---|---|---|---|---|---|---|---|---|---|---|
| *Site* | *ICD code* | *O* | *E* | *O/E* | $\chi^2$ | P | *O* | *E* | *O/E* | $\chi^2$ | P | *O* | *E* | *O/E* | $\chi^2$ | P |
| Stomach | 151 | 15 | 29.2 | 0.51 | 6.90 | <0.01 | 4 | 18.2 | 0.22 | 11.08 | <0.001 | 11 | 11.0 | 1.0 | — | — |
| Lung | 162 | 89 | 92.0 | 0.97 | 0.10 | <0.09 | 66 | 76.1 | 0.87 | 1.34 | <0.3 | 23 | 15.9 | 1.45 | 3.17 | <0.1 |
| Skin | 172–3 | 51 | 43.6 | 1.17 | 1.26 | <0.5 | 31 | 24.7 | 1.26 | 1.61 | <0.3 | 20 | 18.9 | 1.06 | 0.06 | <0.5 |
| Bladder | 188 | 22 | 19.6 | 1.12 | 0.29 | <0.75 | 19 | 15.0 | 1.27 | 1.07 | <0.4 | 3 | 4.6 | 0.65 | 0.56 | <0.5 |
| All | 140–209 | 403 | 422.6 | 0.95 | 0.91 | <0.5 | 201 | 237.0 | 0.84 | 5.72 | <0.025 | 202 | 185.6 | 1.09 | 1.45 | <0.3 |

*Note:* The expected figures are based upon the following:
ICD 151, 162, 172, 188 Registration rates for Scotland for 1968, 1973, and 1977.
ICD 173, Registration rates for England and Wales for 1968, 1973, and 1978.
ICD 140–209 Registration rates for Scotland for 1970, 1973, and 1977.

to the general pattern of the results, and very different from that in females whose observed and expected figures are identical. It also accounts for a large part of the deficiency in the observed figures for all sites among males. No explanation can be offered for this deficit of stomach cancer in the males.

A particular point of interest was whether there was an increase in skin cancer in these patients with psoriasis; both for males and females the ratio of observed to expected cancers is $>1.0$, but in neither sex it is significant.

It is concluded from these results that there is no clear evidence of either a protective effect from the psoriasis in itself, nor a carcinogenic effect from the treatment that the patients have been having.

We are grateful to colleagues at the Scottish Office Computer Services who prepared the files and programmes for the linkage exercises, the Registrar General for Scotland for permission to access the Scottish mortality data, Dr M. Heasman for advice, and Mrs J. Folwell for secretarial help.

**References**

CHAPMAN, P.H., RAWLINS, M.D. & SHUSTER, S. (1979). Activity of aryl hydrocarbon hydroxylase in psoriatic skin. *Lancet*, **i**, 297.

CHAPMAN, P.H., KERSEY, P.J., KEYS, B., SHUSTER, S. & RAWLINS, M.D. (1980). Generalised tissue abnormality of aryl hydrocarbon hydroxylase in psoriasis. *Br. Med. J.*, **281**, 1315.

HALPRIN, K.M. (1980). Psoriasis, skin cancer, and PUVA. *Am. Acad. Dermatol.*, **2**, 334.

HEASMAN, M.A. & CLARKE, J.A. (1979). Medical record linkage in Scotland. *Hlth. Bull.*, **37**, 97.

HILL, I.D. (1972). Computing man years at risk. *Br. J. Prev. Soc. Med.*, **26**, 132.

MORGAN, R.W. (1979). Skin cancer after PUVA treatment for psoriasis. *N. Engl. J. M.*, **301**, 554.

PEMBROKE, A.C., HEHIR, M.E., DU VIVIER, A.W.P. & MARTEN, R.H. (1979). Photochemotherapy and risk of skin cancer. *Lancet*, **i**, 1299.

RAWLINS, M. & SHUSTER, S. (1982). Aryl hydrocarbon hydroxylase in psoriasis. *Lancet*, **ii**, 271.

SEVILLE, R. (1980). Psoriasis. *Medicine*, **30**, 1545.

SHUSTER, S., CHAPMAN, R.H. & RAWLINGS, M.D. (1979). Psoriasis and cancer. *Br. Med. J.*, **i**, 941.

STERN, R.S., THIBODEAU, L.A., KLEINERMAN, R.A., PARRISH, J.A. & FITZPATRICK, T.B. (1979). Risk of cutaneous carcinoma in patients treated with oral methoxysalen photochemotherapy for psoriasis. *N. Engl. J. Med.*, **300**, 809.

STERN, R.S., PARRISH, J.A. & FITZPATRICK, T.B. (1980*a*). Response (to HALPRIN, 1980). *Am. Acad. Dermatol.*, **2**, 337.

STERN, R.S., ZIERLER, S. & PARRISH, J.A. (1980*b*). Skin carcinoma in patients with psoriasis treated with topical tar and ultraviolet radiation. *Lancet*, **1**, 732.